# Study loading capacties of air pollutant emissions for developing countries: a case of Ho Chi Minh City, Vietnam

**DOI:** 10.1038/s41598-020-62053-4

**Published:** 2020-04-02

**Authors:** Bang Quoc Ho, Khue Hoang Ngoc Vu, Tam Thoai Nguyen, Hang Thi Thuy Nguyen, Dung Minh Ho, Hien Nhu Nguyen, Thuy Thi Thu Nguyen

**Affiliations:** Institute For Environment and Resources, Vietnam National University in Ho Chi Minh City, Ho Chi Minh City, Vietnam

**Keywords:** Environmental sciences, Atmospheric chemistry

## Abstract

Ho Chi Minh City (HCMC) is one of the cities in developing countries where many concentrations of air pollutants exceeded the Vietnam national technical regulation in ambient air quality including TSP, NO_x_, Ozone and CO. These high pollutant concentrations have destroyed the human health of people in HCMC. Many zones in HCMC can’t receive more air pollutants. The objectives of this research are: (i) Air quality modeling over HCMC by using the TAPM-CTM system model by using a bottom up air emission inventory; and (ii) Study loading capactities of air pollutant emissions over Ho Chi Minh City. Simulations of air pollution were conducted in Ho Chi Minh City (HCMC), the largest city of Vietnam by using the TAPM-CTM model. The model performance was evaluated using observed meteorological data at Tan Son Hoa station and air quality data at the Ho Chi Minh City University of Science. The model is then applied to simulate a retire 1-year period to determine the levels of air pollutants in HCMC in 2017, 2025 and 2030. The results show that the highest concentrations of CO, NO_2_, and O_3_ in 2017 exceeded the National technical regulation in ambient air quality (QCVN 05:2013) 1.5, 1.5, and 1.1 times, respectively. These values also will increase in 2025 and 2030 if the local government does not have any plan for the reduction of emissions, especially, SO_2_ in 2030 also will be 1.02 times higher than that in QCVN 05:2013. The emission zoning was initially studied by calculating and simulating the loading capacities of each pollutant based on the highest concentration and the National technical regulation in ambient air quality. The results show that the center of HCMC could not receive anymore the emission, even needs to reduce half of the emission. Under the easterly prevailing wind in the dry season, the high pollution was more likely to be experienced in the west of Ho Chi Minh. In contrast, the eastern regions were the upwind areas and the pollutants could transport to the downwind sectors. It was recommended that the best strategy for emission control in HCMC is avoiding industrial and urban development in the upwind areas to achieve better air quality for both areas. In the case of necessity to choose one area for development, the downwind sector is preferred. The results show that TAPM-CTM performed well as applied to simulate the air quality in HCMC and is a promising tool to study the emission zoning.

## Introduction

Ho Chi Minh City (HCMC), the largest city in Vietnam with its position as the political, economic, scientific and cultural center of the country, is located at 10^o^45′N and 106^o^45′E in the south-eastern region of Vietnam. HCMC’s economic growth rate has been skyrocketing in recent years. The population of HCMC is 8.6 million people, the number of private vehicles is about 9 million units. Currently, there are 19 manufacturing and industrial zones, 30 industrial clusters on an area of 1,900 ha, and numerous factories and enterprises located separately in HCMC^1,2^. All those activities could release a huge amount of air pollutants into the atmosphere.

Urbanization and economic development during the recent period in HCMC have caused numerous environmental and social issues^3^. Many air pollutants in HCMC exceeded the National technical regulation in ambient air quality (QCVN 05:2013, namely QCVN from now) including TSP, NO_x_, and CO^1,4^. These high pollutant concentrations were associated with an increase in the risk of human health in HCMC^5–8^. It is urgent to determine the needed amount of emission reduction and the most polluted areas in the city to build the best abatement strategies for the reduction of emission. This status has raised the concern of scientific communities and policymakers. The combination of scientists and the city’s government is essential to make appropriate planning policies. The environment-responsive strategies for developing economics require a comprehensive understanding of the local environmental conditions. Studies of overall status and forecast of air pollution are very important to implement these strategies. However, these studies in HCMC have still been patchy, quite dated^9,10^ and need to be updated. In addition, the current emission in HCMC has not taken into account the pollutant loading capacities of the atmosphere with the specific condition of the city. Only when determining this capacity for each region, can policymakers be able to localize reasonable emission areas (defined as the emission zoning by us). Therefore, this study aims at (i) Air quality modeling over HCMC by using the TAPM-CTM system model by using a bottom up air emission inventory; and (ii) Study loading capactities of air pollutant emissions over Ho Chi Minh City.

## Materials and Methods

### Meteorological and air quality modelling

The Air Pollution Model (TAPM), an easy-to-use and fast-to-run model which is a feasible tool for meteorological and air pollution simulations, was developed by Commonwealth Scientific and Industrial Research Organization - Commonwealth Scientific and Industrial Research Organization (CRISO) of Australia^11^. TAPM has meteorological and air pollution module, in which the consists of the former are parameterizations for cloud/rain microphysical processes, turbulence closure, urban/vegetative canopy, and soil, and radiative fluxes and those of the later are various sub-modules including the Eulerian Grid Module (EGM), the Lagrangian Particle Module (LPM), the Plume Rise Module (PRM) and the Building Wake Module (BWM). Detail descriptions of the model were described by Hurley *et al*. (2005 and 2008)^11,12^. For simulations that require complex chemical transformation, CRISO developed an enhanced version of TAPM referred to TAPM-CTM^13^. The advances of TAPM-CTM compared to TAPM analyzed thoroughly in the study of Bang *et al*.^14^ in which the prognostic model provides the meteorological fields that drive dispersion of emissions and pollutant concentrations in the chemical transport model CTM. The first version of TAPM model, developed by Peter Hurley *et al*., has been developing since 1999. This model was continuously improved to version 4 in 2008 to fix the problems of the previous version. The validation of TAPM model has been performed through several comparative studies. For example, the comparison between the simulation of air quality for the Port Phillip and the observed values at mornitoring stations of EPA Victotira, Australia^15^, between the silmulation of PM_10_ and the observed values at mornitoring station Christchurch, New Zealand^16^. In recent years, TAPM-CTM has been widey applied for simulating NO, NO_2_, and O_3_ in the Greater Metropolitan Region in New South Wales, Australia^17–21^. This model was applied in HCMC to simulate the photochemical smog in HCMC in 2018^14^.

### Input data

Input data of TAPM-CTM modeling system include two components: (i) the global meteorological data from The Australian Community Climate and Earth-System Simulator (ACCESS) which are available online and can be downloaded via CSIRO’s website, and (ii) air emission inventory data within the region under consideration.

Emission data that have been completed and published were used as input to the air quality model^1^. A comprehensive of Emission Inventory (EI) in 2017 and emission forecast in 2025 and 2030 over HCMC including point, line, area, and biogenic sources were conducted in that study. For line sources, the EMISENS (EMIssion SENSitivity) model, a model combining the top-down and bottom-up approaches, was applied. For the other sources (point, area and biogenic sources), a emission factor approach and survey data, was used to calculate air emission. The air emission forecast until 2030 was calculated by using the data of strategies and plans for the socio-economic development of HCMC in the period until 2030. The EI was calculated for NO_x_, SO_2_, CO, NMVOC, TSP, and CH_4_ with a temporal resolution of one hour and a spatial resolution of 2,5 km × 2,5 km. The total emission from four main sources of air pollutants in HCMC in 2017 and 2030 is presented in Table 1 ^1^. In general, emission in 2030 are expected to be significantly higher compared to 2017.

**Table 1:** Total emissions in HCMC (tons/year) in HCMC in 2017, 2025 and 2030^1^.

Year	Emission (tons/year)					
	NOx	CO	SO_2_	NMVOC	TSP	CH_4_
2017	50,386	3,533,982	12,919	602,625	8,545	15,957
2025	73,191	5,192,753	18,746	880,869	11,090	21,873
2030	81,824	5,863,397	21,071	993,562	12,175	24,068

### Modelling domains

**Figure 1:**
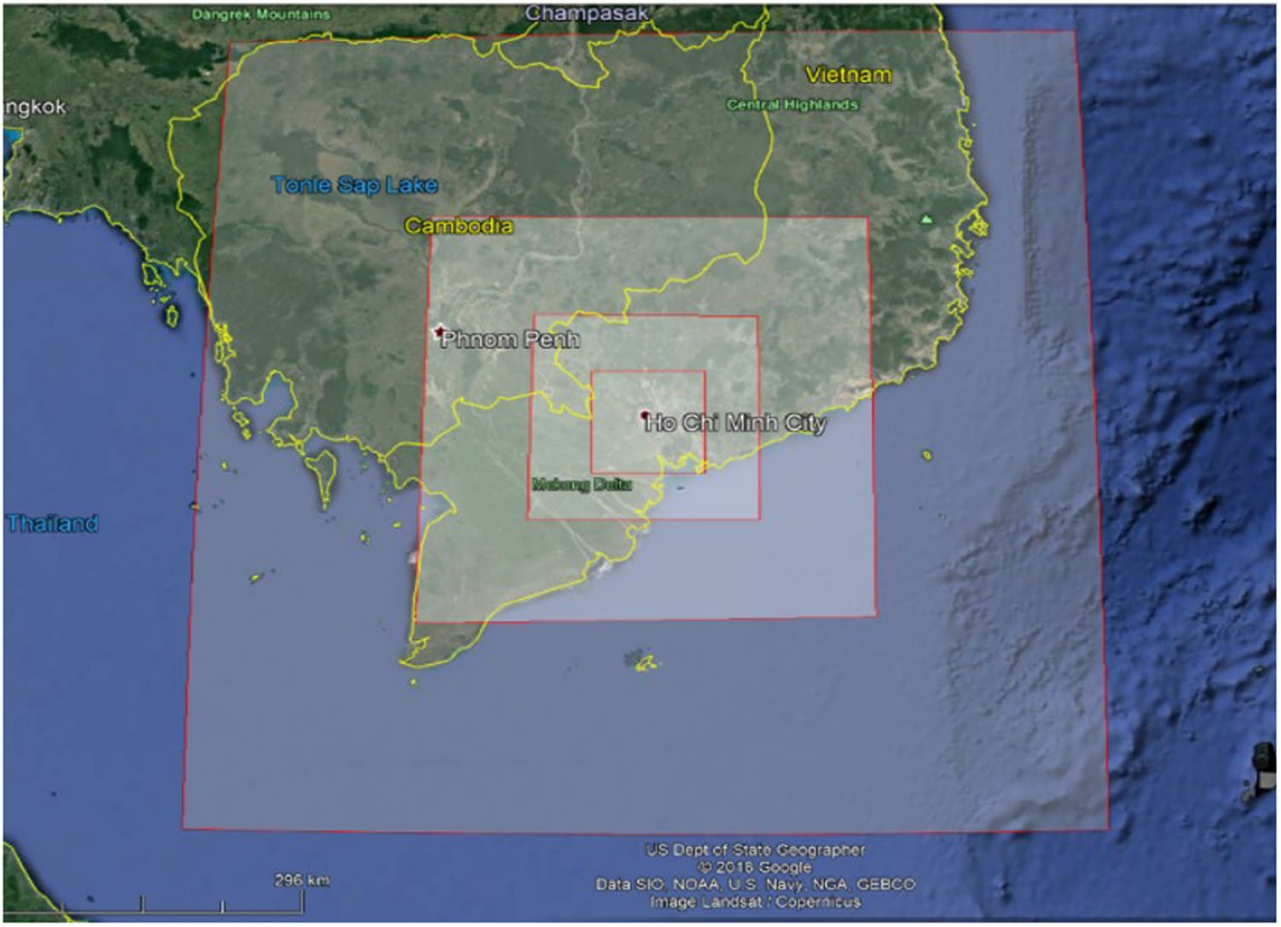
TAPM-CTM modelling domains (D1, D2, D3 and D4).

Four domains were configured in this study (Fig. 1) including (i) the outer most domain D1 characterizing the south of Vietnam (800 km × 800 km), (ii) the wider domain D2 characterizing Mekong Delta (400 km × 400 km), (iii) the domain D3 characterizing HCMC and some neighboring provinces (200 km × 200 km), and (iv) the subdomain D4 characterizing the main part of HCMC (100 km × 100 km). Each domain was 40 by 40 grid with the resolution was 20, 10, 5, and 2.5 km for D1, D2, D3, and D4, respectively. The three outer domains (D1, D2, D3) only simulated meteorology, the interior domain (D4) simulated both meteorology and chemical processes. The simulation results of the coarser revolution were the input data for the next inner domain. For instance, the simulation results of D1 were the meteorological boundary conditions for the D2. The size of the inner-most domain (D4) was set to be the same as the HCMC emission inventory domain. The meteorological grids must be greater or equal to the emission grids; therefore, the emission inventory domain was set 90 km by 90 km with 35 grids and the grid resolution was 2.5 km.

### Model evaluation

Statistical measures for model evaluation were proposed by several researchers in order to evaluate the TAPM model’s performance^12^ and this approach also was used in our study by comparing the modeled data with observations from the field. In which meteorological data at Tan Son Hoa station and air quality data at Nguyen Van Cu station were used to assess the TAPM and CTM model, respectively. More specifically, statistical parameters including Pearson correlation coefficient (R) between observed (O) and predicted (P) values, mean value, standard deviation, minimum value (min), and maximum value (max) were used in this study.

### Loading capacties

A technical approach, modeling tool, was utilize to calculate and simulate the loading capacities of each pollutant in HCMC. Based on emission inventories and optimization of maximum total emission, under the criterion that target pollutant concentrations at monitoring sites meet national standards, the loading capacities of each pollutant were determined. This approach was also applied to calculate atmospheric environmental capacities in several studies^22^.

## Results and Discusion

### Model calibration and validation

#### Performance of Meteorological model - TAPM

Meteorological observation data from Tan Son Hoa station (10.7969^o^N, 106.6668^o^E) were used to calibrate and validate the performance of the TAPM-CTM prediction. Surface temperature and wind speed were parameters that were evaluated with observed data in this study. Statistical parameters for hourly of predicted (P) and observed (O) temperature and wind speed at Tan Son Hoa station are presented in Table 2.

**Table 2:** Statistical parameters for hourly of modeling (P) and monitoring (O) temperature and wind speed at Tan Son Hoa station.

	February 2017		June 2017		February 2017		June 2017	
	Temperature (^o^C)				Wind speed (m/s)			
	P	O	P	O	P	O	P	O
Mean	27.1	28.1	31.6	30.2	2.2	1.8	2.4	1.7
Standard deviation	3.9	2.8	3.7	2.7	1.0	1.0	1.1	0.9
Min	18.9	22.2	23.7	24.1	0.2	0.0	0.5	0.0
Max	34.0	34.5	37.3	36.6	5.1	7	5.5	7.0

**Figure 2:**
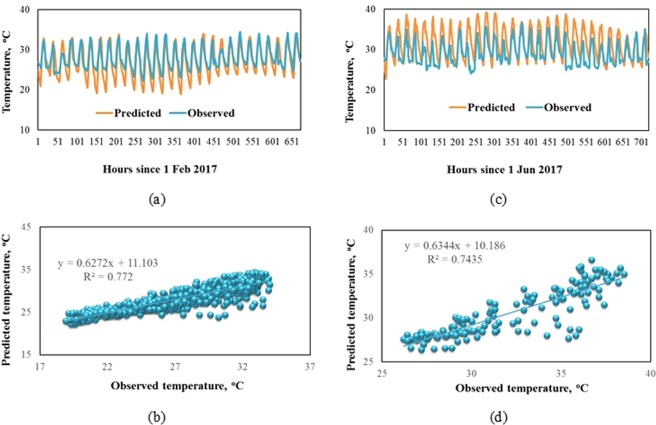
Predicted and observed surface temperature at Tan Son Hoa station: (**a**) Time series of observed and predicted surface temperature in February 2017; (**b**) Regression analysis of observed (x-axis) and predicted (y-axis) surface temperature in February 2017; (**c**) Time series of observed and predicted temperature in June 2017; (**d**)) Regression analysis of observed (x-axis) and predicted (y-axis) surface temperature in Jun 2017.

The predicted temperature for January to December 2017 correlated well with observed temperature as shown in the time series plots and the regression analysis in Fig. 2, in which February and June represent for the dry (Fig. 2a,b) and the rainy season (Fig. 2c,d), respectively.

Figure 2 and Table 2 shows that the model predicted well the surface temperature with 1.0 and 1.4 ^o^C difference in February and June, respectively. Figure 2 shows that the value R between simulation and monitoring for temperature 0.77 in February 2017 and 0.74 in June 2017 which is a good performance for temperature modeling. However, TAPM slightly underestimated the temperatures during the dry season but a little overestimated during the rainy season. This result also agreeded with the findings in Matthaios’s study in 2018 about the evaluation of TAPMP model over a mountainous complex terrain industrial area^23^.

TAPM also simulated well the surface wind speed in the study area, with the mean values of both predicted and observed wind speed were approximate 2 m/s during the study period. However, it slightly overestimated the wind speed comparing with observed value and with 0.4 and 0.7 m/s difference in February and June, respectively, which were the same ranges as those in Matthaios’s study^23^.

#### Performance of Air Quality model - CTM

**Figure 3:**
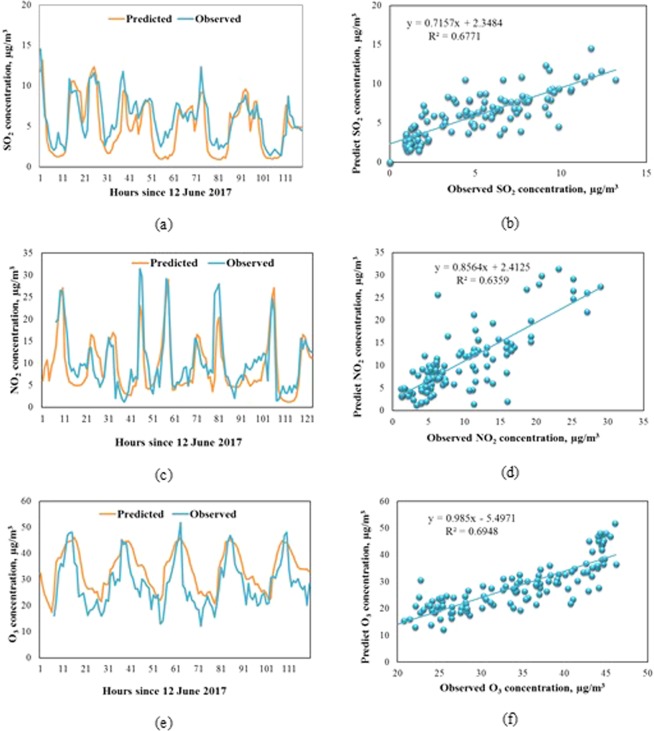
Predicted and observed air quality at Nguyen Van Cu station from 12 to 17 June 2017: (**a**) Time series of observed and predicted SO_2_ concentration; (**b**) Regression analysis of observed (x-axis) and predicted (y-axis) SO_2_ concentration; (**c**) Time series of observed and predicted NO_2_ concentration; (**d**)) Regression analysis of observed (x-axis) and predicted (y-axis) NO_2_ concentration; (**e**) Time series of observed and predicted O_3_ concentration; (**b**) Regression analysis of observed (x-axis) and predicted (y-axis) O_3_ concentration.

The simulation results of air quality from the CTM model were validated with observed values at 03 locations: the University of Natural Science located at 227 Nguyen Van Cu street (10.762549^o^N, 106.682428^o^E) in HCMC, the Tan Binh industrial Zone (10.804863^o^N, 106.637664^o^E) and Linh Trung Exporting Zone (10.855993^o^N, 106.799454^o^E). Figure 3 and Table 3 present the performance of SO_2_, NO_2_, and O_3_ from 12^th^ to 17^th^ June 172017.

**Table 3:** Statistical parameters for hourly of predicted (P) and observed (O) SO_2_, NO_2_, and O_3_ at Nguyen Van Cu station.

	SO_2_		NO_2_		O_3_	
	P	O	P	O	P	O
R^2^	0.677		0.636		0.695	
Mean	4.9	5.9	9.4	10.5	34.0	28.5
Standard deviation	3.3	2.8	6.4	6.9	7.9	9.0
Min	0.9	1.4	1.2	1.2	17.5	12.2
Max	13.2	14.5	29.0	31.4	46.2	51.8

Figure 3 and Table 3 show that the model predicted quite well the air quality with the correlations (R^2^) between hourly concentrations of predicted and those of observed values were always nearly 0.7 for the fist location at the University of Natural Science, for the second and third locations have the correlations (R^2^) between hourly concentrations of predicted and those of observed values were higher than 0.7. When considering the time series of observed and predicted pollutant concentration, simulation results tend to be similar to actual monitoring results with maximum and minimum values. The model predicted well the concentration of SO_2_ and NO_2_ with 1.0 and 1.1 µg/m^3^ difference, respectively. However, CTM slightly overestimated the concentration of O_3_. This result was different from that of Bang’s study about the simulation of O_3_ in HCMC in 2015 (the research in 2015, air emission inventory results were used from top-down approach, therefore the results of modeling in 2015 have more uncertainty than the current research using detail air emission inventory), in which the authors found that the model overall underpredicted the ozone prediction at Nguyen Van Cu site^1^. This difference could be understood because the period when Bang *et al*. compared the observed and predicted ozone concentrations was the dry season when high pollution usually happens in HCMC^1^; whereas the comparison of those in this study was the wet season when the lower concentrations of pollutants are found. This indicated that the TAPM-CTM model tends to underestimate the concentrations in the highly polluted periods and overestimate in the little polluted ones.

In addition, The monitoring site that we used for validation models for the metrological condition was different from that for the air pollutants. However it doesn’t affect the research because the TAPM-CTM could provide the simulation results for each point. We can select any point in our domain for meteorological and air quality validation.

### Simulation of air quality in HCMC

#### Simulation of air quality in HCMC in 2017

After evaluating the model, simulation the air quality for each month in 2017 was conducted to determine the areas and period having a high concentration of pollutants. The simulation results included all of one-hour, eight-hour, and twenty four-hour average concentration of each pollutant, in which CO, NO_2_, and SO_2_ were the primary pollutant group and O_3_ was the secondary pollutant group.

**Figure 4:**
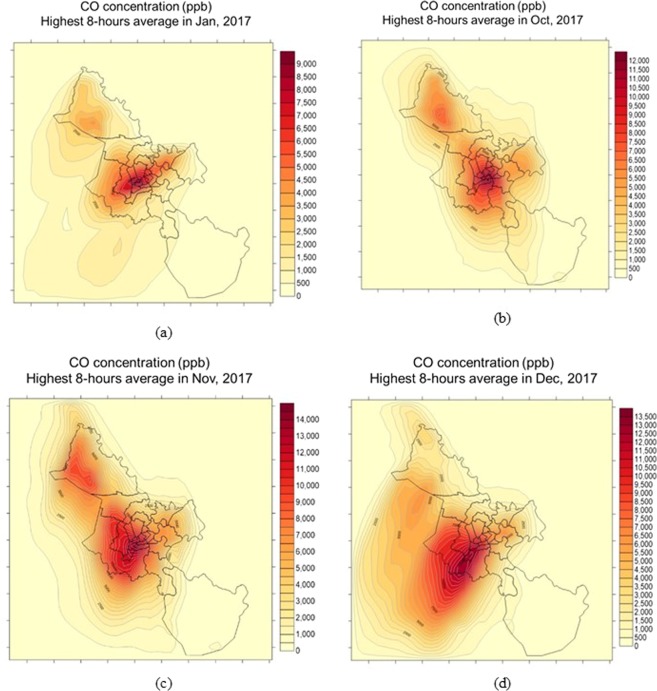
The high concentrations of CO: (**a**) eight-hour average concentration in Jan 2017; (**b**) eight-hour average concentration in Oct 2017, (**c**) eight-hour average concentration in Nov 2017; (**d**) eight-hour average concentration in Dec 2017.

The primary pollutants. The high concentrations of CO are presented in Fig. 4, in which, Fig. 4a is the eight-hour average concentration in Jan 2017, Fig. 4b is the eight-hour average concentration in Oct 2017, Fig. 4c is the eight-hour average concentration in Nov 2017 and Fig. 4d is the eight-hour average concentration in Dec 2017.

Generally, the one-hour average concentration of CO did not exceed the standard of QCVN, in which the concentrations of CO from February to August were relatively lower than those in remaining months. However, the eight-hour average concentrations in October, November, December, and January reached from 12,000 ppb to 14,000 ppb (about 13,560–15,820 µg/m^3^) exceeding QCVN (10,000 µg/m^3^) from 1.3 to 1.5 times. The highest one-hour average concentration of CO was 26,000 ppb (29,380 µg/m^3^) in November 2017 that was approximate the standard of QCVN (30,000 µg/m^3^). In this day, the eight-hour average concentration of CO reached 14,000 ppb (15,820 µg/m^3^) that was 1,5 times higher than the standard of QCVN. The highest concentration of CO also was about 2 time higher than that previous studies^10,11^ that could be due to their usage of different model and different EI method. The more comprehensive calculation of EI including line, area, point, and biogenic sources were conducted in our study. In which, the traffic sources consisted both on-road and non-road source having the airport, seaport, and bus and railway station, the area source included households, restaurants, gas stations, constructions sites, photocopy stores, construction material stores, pagodas, and garages^1^. The plume of CO was located in the center of HCMC where CO was mainly emitted^1^.

**Figure 5:**
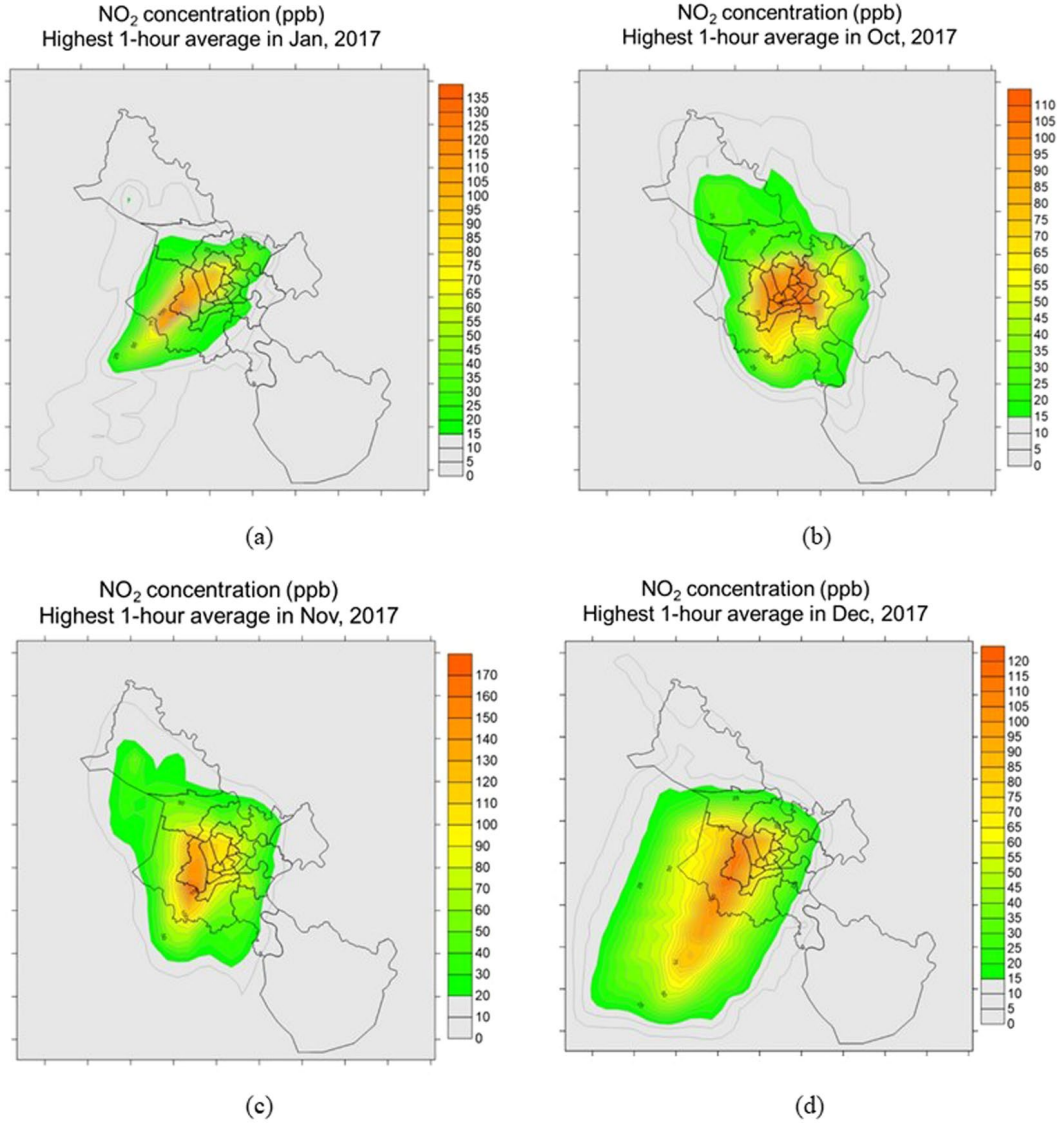
The high concentrations of NO_2_: (**a**) eight-hour average concentration in Jan 2017; (**b**) eight-hour average concentration in Oct 2017, (**c**) eight-hour average concentration in Nov 2017; (**d**) eight-hour average concentration in Dec 2017.

The high concentrations of NO_2_ are presented in Fig. 5, in which, Fig. 5a is the one-hour average concentration in Jan 2017, Fig. 5b is the one-hour average concentration in Oct 2017, Fig. 5c is the one-hour average concentration in Nov 2017 and Fig. 5d is the one-hour average concentration in Dec 2017.

Similar with CO, the high one-hour average concentration of NO_2_ occurred in January, October, November, and December that were 135; 110; 170; and 120 ppb, respectively. The hourly concentrations of other months were from 42 to 65 ppb. The highest hourly concentration of NO_2_ was recorded in 17^th^ November 2017 with the value of 170 ppb (equivalent 200 µg/m^3^) exceeding QCVN (10,000 µg/m^3^) 1.5 times. The highest concentration of NO_2_ also was from 1.3 to 1.9 time higher than that previous studies^6,8^ that could be explained by using a different model and different EI method mentioned above. The plume of NO_2_ also located in the center of HCMC having the dense transportation system and seaport^1^.

**Figure 6:**
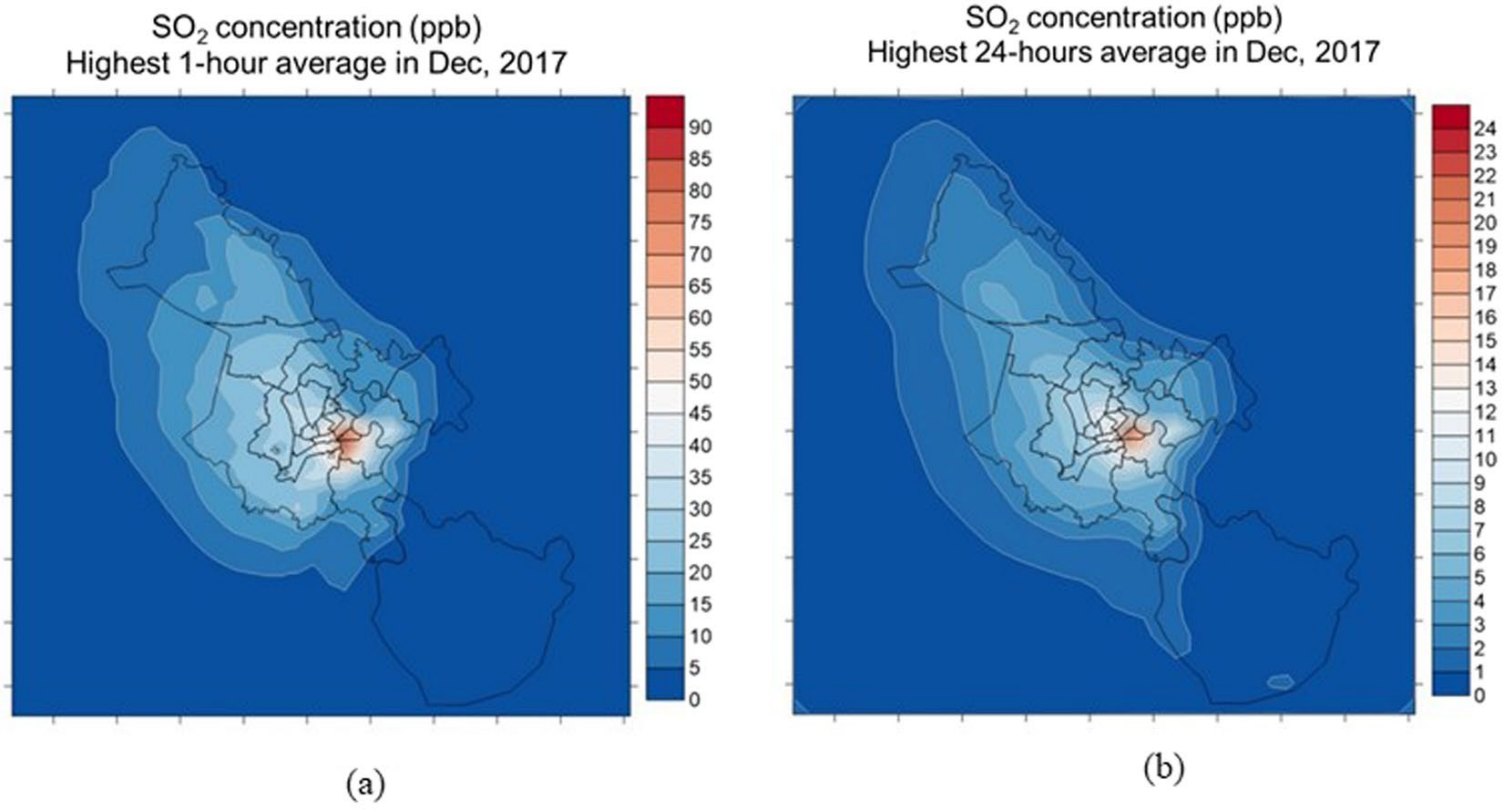
The high concentrations of NO: (**a**) one-hour average concentration in Dec 2017; (**b**) 24-hour average concentration in Dec 2017.

The highest concentrations of SO_2_ are presented in Fig. 6a, in which, Fig. 6a is the one-hour average concentration in Dec 2017, Fig. 6b is the one24-hour average concentration in Dec 2017.

Generally, the all highest concentrations of SO_2_ was lower than the standard in QCVN, in which the highest one-hour average concentration and 24-hour average concentration was 90 and 24 ppb (equivalent 232 and 61.9 µg/m^3^), respectively. The highest concentration of SO_2_ in this study also was about 1.5 times higher than that in Dung’s study^7^ that was similar to other primary pollutants above. The plume of SO_2_ located at district 2 and district 4 having many seaports, other urban and the suburban area was lower than 40 ppb and 25 ppb, respectively.

**Figure 7:**
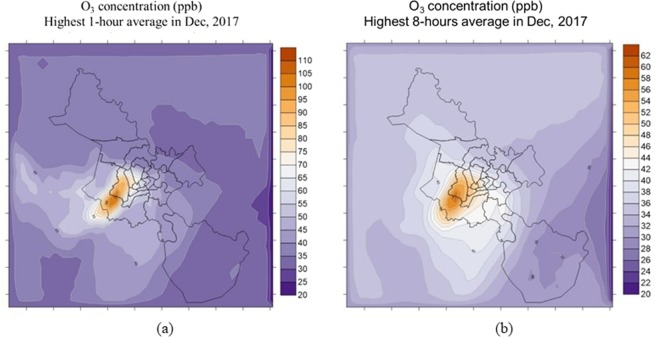
The highest concentrations of ozone: (**a**) one-hour average concentration; (**b**) eight-hour average concentration.

The secondary pollutant. The highest concentrations of ozone are presented in Fig. 7, in which, Fig. 7a is the one-hour average concentration and Fig. 7b is the eight-hour average concentration.

The high hourly ozone concentrations were from 53 to 110 ppb, in which the concentrations in the last months of the year were higher than those of the first months. The highest concentrations were recorded in 13^th^ December 2017 that was the beginning of the dry season. The one-hour average concentration of ozone reached 110 ppb (equivalent to 220 µg/m^3^) exceeding about 1.1 times of the National technical regulation in ambient air quality standard (QCVN). The eight-hour average concentration of ozone also was recorded in this day with the value of 62 ppb (equivalent to 124 µg/m^3^) that was higher than that in QCVN (120 µg/m^3^). The ozone plume tended to move southwestward of the city, only a few times in May and August, the plume pushed to the northwest or northeast of HCMC. The results of ozone simulation in this study were compared with those in other studies presented in Table 4.

**Table 4:** Comparison results of ozone simulations in HCMC with other studies.

Studies	Conducted period	EI method	Model	Prevailing wind direction	Plume direction	Maximum concentration
(Nghiem, 2009)^24^	Three days in Jan 2005	Top-down approach, data were collected from satellite image (MODIS) in 2005	CMAQ-MM5	Northerly	Southwest	50 ppb
(Dung, 2009)^10^	Two days in Jan 2006	Top-down and bottom-up approach, data were calculated for on-road, industrial, and domestic sources	FVM-TAPOM	Northerly	Southwest	75 ppb
(Bang, 2011)^11^	Three days in Feb 2006	Top-down and bottom-up approach, data were calculated for on-road, industrial, and domestic sources	FVM-TAPOM	Easterly	Northern and Northwestern	150 ppb
(Bang, 2018)^15^	Entire one-month period in Jan 2015	Top-down and bottom-up approach, data were calculated for traffic, industrial, household, and biogenic sources	TAPM-CTM	Northerly	Southwest	65 ppb
This study	Entire 1-year period in 2017, the maximum concentration occurred in Dec 2017	Top-down and bottom-up approach, data were calculated for line source (including on-road and non-road such as airport, seaport, and bus station; area sources including household, restaurants, gas station, etc.; point sources and biogenic sources	TAPM-CTM	Northerly	Southwest	110 ppb

Table 4 shows that the dominant plume pattern of ozone in this study was similar to that in other studies; however, there was a difference in the maximum concentration of ozone in these studies. The maximum concentration in this study was about 2 times higher than that in other studies except for Bang’s study in 2011. This difference could be explained by using different models and input data explained above.

#### Air pollution forecast for Ho Chi Minh City in 2025 and 2030

Based on the data of strategies and plans for socio-economic development of HCMC in the period 2025 and 2030, the simulations of air quality for 2025 and 2030 were conducted in order to forecast how the future growth impact to the air quality.

For the year of 2025, if HCMC continues to develop the socio-economic plan, the air quality will become worse. The one-hour and eight -hour average concentration of ozone will be 230 µg/m^3^ and 144 µg/m^3^ exceeding the standard of QCVN 1.15 and 1.20 times, respectively. The one-hour and eight -hour average concentration of CO will reach 31,640 µg/m^3^ and 18,080 µg/m^3^ exceeding the standard of QCVN 1.05 and 1.80 times, respectively. The one-hour average concentration of NO_2_ also will be 297 µg/m^3^ exceeding the standard of QCVN 1.50 times. Only SO_2_ concentration will meet the QCVN standard.

According to the development plan of HCMC by 2030, the air pollution situation will become more serious. The one-hour and eight-hour average concentration of CO and O_3_ will be 1.7 and 1.15 times higher than the standard of QCVN, respectively. The highest hourly concentration of NO_2_ will reach 180 ppb (equivalent 334 µg/m^3^) exceeding the QCVN 1.57 times. Especially, the highest hourly concentration of SO_2_ also will be 1.02 times higher QCVN.

### Loading capacities of air emissions in HCMC

It is obvious that if HCMC does not have a good plan to develop the social and economics, the air pollution situation in this city will become worse. Determining the pollutant loading capacities for each small area could help policy-makers to improve efficiency in building the abatement strategies. Therefore, in this study, we initially studied the emission zoning (or loading capactities) for HCMC. From the simulation results of air quality above, areas were zoned by comparing with the QCVN. For areas with lower concentrations than QCVN, we increased the emission for each grid until to meet the standard. For areas with higher than QCVN, we reduced the emission for each grid until to meet the standard. In the calculation process, we also took into account the long-range transport based on the meteorological simulation results. For example, if the polluted area (A) was caused by emissions from the neighboring area (B), the B area would be diminished the emissions to reduce pollutant concentration at A area. We also considered the ability to increase emissions at A to determine whether the pollutant concentration in this area to increase or not.

After calculating and simulating, we found that in order to control the air quality to meet the standard of QCVN, HCMC needs to reduce emissions for NO_2_ and CO 1.58 times, equivalent to 58%. Beside, HCMC also has the ability to receive 1.51 times of additional SO_2_ emissions, equivalent 51% in the future to ensure that the SO_2_ concentration will meet the standards.

**Figure 8:**
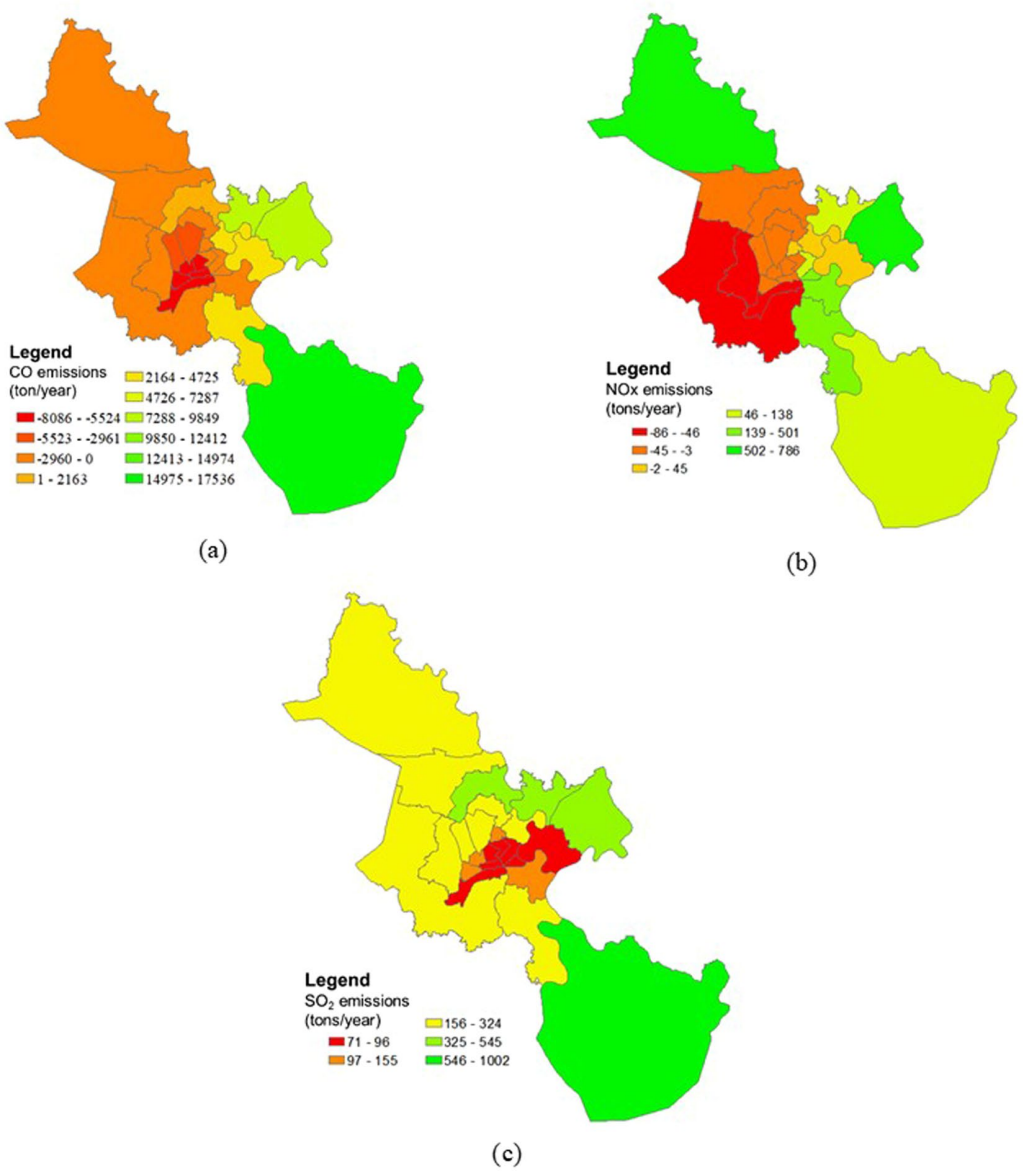
The emission zoning: (**a**) the emission zoning of CO; (**b**) the emission zoning of NO_2_; (**c**) the emission zoning of SO_2_.

The emission zoning of each pollutant are presented in Fig. 8, in which, Fig. 8a is the emission zoning of CO, Fig. 8b is the emission zoning of NO_2_, and Fig. 8c is the emission zoning of SO_2_.

For CO, it is necessary to reduce emissions in the central districts and the northern, the western suburban districts. Specifically, the areas that need to reduce the most CO emission are District 10, District 11, District 5, District 6 and District 8, which require a reduction of 5,500–8,000 tons/year.km^−2^; Tan Binh and Tan Phu districts need to reduce 3,000 to 5,500 tons/year.km^−2^; District 7, District 4, District 1, District 3, Phu Nhuan District Cu Chi, Hoc Mon, Binh Chanh and Binh Tan, and Go Vap are necessary to reduce emissions by 3,000 tons/year.km^−2^. The districts could receive additional CO emissions as follows: District 12 might increase 2,100 tons/year.km^−2^, Binh Thanh, District 2, Nha Be could increase to 7,200 tons/year.km^−2^, Thu Duc and District 9 might increase by 9,800 tons/year.km^−2^, and Can Gio area could increase to 17,500 tons CO/year.km^−2^. However, Can Gio is the city’s biological reserve area; therefore, the emission addition should be considered.

For NOx, the western areas of the city including District 8, Binh Tan and Binh Chanh need to be reduced from 46–86 tons/year.km^−2^; District 10, District 3, District 5, District 6, District 11, Tan Phu, Tan Binh, Go Vap, District 12, and Hoocmon need to reduce from 3 to 45 tons/year.km^−2^. The remaining areas can receive NO_x_ additional emissions with the amount of 45–500 tons/year.km^−2^, in which Cu Chi and District 9 are the two areas that can receive more NO_x_ emission. The maximum possible level of these areas could be reached about 500–786 tons/year.km^−2^.

Generally, SO_2_ concentration is lower than the standard of QCVN; therefore, SO_2_ could be added to the atmosphere as follows: areas of downtown districts such as Districts 1, District 3, District 4, District 10, District 5, District 8, District 2 could be increased about 71–96 tons/year.km^−2^; District 7, Phu Nhuan, District 11, and District 6 might add 97–155 tons/year.km^−2^; the suburban districts could receive from 156 to 545 tons/year.km^−2^; Can Gio might receive the largest amount of SO_2_ being from 546–1,002 tons/year.km^−2^.

For VOCs, the current emission was maintained because VOCs relate to O_3_ generation reactions. With the current scenario, the highest O_3_ concentration already exceeded QCVN. After simulating the NO_x_ reduction scenario by keeping the VOCs emission, the O_3_ concentration also reduced to meet the standard. Therefore, it is not advisable to add VOC’s emission to the central districts and the western districts of HCMC because these areas had eight-hour average concentration that approximates the QCVN.

In general, the central areas of HCMC need to reduce half emissions. The city’s government need to consider reducing the number of private vehicles in the city because this is the main source of air^1^. The areas of Binh Chanh and Binh Tan districts have lower emissions than the central areas. However, these areas are influenced by the pollutant plumes from the center due to the wind from the East Sea. In November and December, this area often has higher concentrations of pollutants exceeding the QCVN. In contrast, the air polluatants in Thu Duc and District 9 could transport to the downwind sector because of wind from the East Sea could blow pollutant plumes from these areas to the west and northwest of the city. Therefore, HCMC city should restricted industrial and urban development in the these areas in term of emission control. If it is necessary to choose one area for development, the downwind sector is preferred. Can Gio area is the place with the lowest pollutant concentration and has the ability to receive the highest emissions. However, this area is the biosphere reserve of the city. Therefore, this area should be kept the status without developing the urbanization and industry. This research did not take into account the transboundary transportation of air pollutants. The further studies including both the local and long-range sources need to be considered to get the more realistic simulation results of air quality in HCMC.

## Conclusions

An air quality simulation for CO, NO_2_, SO_2_, and O_3_ over HCMC was conducted in this paper by using the TAPM-CTM model. Overall, the simulation for air quality performed reasonably well in predicting the pollutant levels in 2017. Based on the simulation results of the current status of air quality, it is obvious that the period of high pollution usually is in the last months of the year. This time is the late of the rainy season with low rainfall and the weather is unfavorable to diffuse pollutants, resulting in the highest one-hour average concentration for NO_2_ and O_3_, the highest eight-hour average concentration for CO exceeding the standard of QCVN 1.5, 1.1, and 1.5 times, respectively. Only the concentration of SO_2_ was lower than the standard.

The air quality forecasts for HCMC by 2025 and 2030 also were simulated based on the data of strategies and plans for socio-economic development of the city. The results showed that both the highest one-hour and eight-hour average concentration of O_3_, CO, and NO_2_ were higher than QCVN by the year 2025 and 2030. Especially, if HCMC continues to develop the socio-economic plan, the highest hourly concentration of SO_2_ also will be 1.02 times higher QCVN by the year of 2030.

The emission zoning were initially studied by calculating and simulating the loading capacities of each pollutant. Generally, the downtown HCMC need to reduce about half of emission; therefore, city authorities should consider stopping the development of industry and urbanization of this area. In addition, it is necessary to reduce the number of private vehicles in this area because this is the main source of pollution. The eastern areas of the city also need to limit the development of industry and urbanization due to the wind from the East Sea blows the pollutants to the west and the north. In the case of necessity to choose one area for development, the downwind sector is preferred. We also proposed keeping the status of Can Gio area although this area could receive more additional emission because Can Gio is the biosphere reserve of the city in particular and of the country in general.
